# Identifying the views of adolescents in five European countries on the drivers of obesity using group model building

**DOI:** 10.1093/eurpub/ckaa251

**Published:** 2021-02-20

**Authors:** Natalie Savona, Talia Macauley, Anaely Aguiar, Anna Banik, Monika Boberska, Jessica Brock, Andrew Brown, Joshua Hayward, Helene Holbæk, Ana Isabel Rito, Sofia Mendes, Fredrik Vaaheim, Marloes van Houten, Gerlieke Veltkamp, Steven Allender, Harry Rutter, Cecile Knai

**Affiliations:** 1 Health Services Research and Policy, London School of Hygiene and Tropical Medicine, London, UK; 2 Department of Geography, System Dynamics Group, University of Bergen, Bergen, Norway; 3 CARE-BEH Center for Applied Research on Health Behavior and Health, SWPS University of Social Sciences and Humanities, Wroclaw, Poland; 4 School of Health Sciences, City University of London, London, UK; 5 Global Obesity Centre, Institute for Health Transformation, Deakin Unive rsity, Geelong, Victoria, and Australia; 6 Department of Nutrition, University of Oslo, Oslo, Norway; 7 Centre for Studies and Research on Social Dynamics and Health—CEIDSS, Lisbon, Lisbon, Portugal; 8 Press, Oslo, Norway; 9 Department of Sociology, University of Amsterdam, Amsterdam, The Netherlands; 10 Department of Social and Policy Sciences, University of Bath, Bath, UK

## Abstract

**Background:**

To make effective progress towards a global reduction in obesity prevalence, there needs to be a focus on broader structural factors, beyond individual-level drivers of diet and physical activity. This article describes the use of a systems framework to develop obesity prevention policies with adolescents. The aim of this research was to use the group model building (GMB) method to identify young people’s perceptions of the drivers of adolescent obesity in five European countries, as part of the EU-funded Co-Create project.

**Methods:**

We used GMB with four groups of 16–18-year-olds in schools in each of the five European countries (The Netherlands, Norway, Poland, Portugal and the UK) to create causal loop diagrams (CLDs) representing their perceptions of the drivers of adolescent obesity. The maps were then merged into one, using a new protocol.

**Results:**

Two hundred and fifty-seven participants, aged 16–18 years, engaged in 20 separate system mapping groups, each of which generated 1 CLD. The findings were largely congruent between the countries. Three feedback loops in the merged diagram particularly stand out: commercial drivers of unhealthy diets; mental health and unhealthy diets; social media use, body image and motivation to exercise.

**Conclusions:**

GMB provides a novel way of eliciting from young people the system-based drivers of obesity that are relevant to them. Mental health issues, social media use and commercial practices were considered by the young people to be key drivers of adolescent obesity, subjects that have thus far had little or no coverage in research and policy.

## Introduction

Adolescent obesity not only cause problems in terms of physical and mental health in young people but also carries the likelihood of continuing into adulthood, thus increasing the risk of a range of poor health outcomes.^1^ To date, there has been relatively limited research and policy focus on *adolescent* obesity yet one in seven 15 years olds in Europe is overweight or obese^2^ and by 2025, one in every five children is expected to be overweight.^3^ Overweight and obesity in youth are the main predictors of overweight/obesity and related chronic disease risk in adulthood and throughout the life-course; they increase the risk of many non-communicable diseases, accounting for around 60% of the risk of developing Type 2 diabetes, over 20% of the risk of hypertension and coronary-heart disease and 10–30% of the risk for several cancers.^4^ Meanwhile, interventions designed to reduce obesity prevalence that do target this group are predominantly focused on factors designed to influence individuals’ beliefs, skills and behaviours rather than environmental drivers of diet and physical activity.^5^ A number of behavioural factors such as time spent using electronic screen devices, meal frequency and physical activity are known contributors to body weight^6^ but less is known about the drivers of these factors and how they are interconnected. Persistently high obesity prevalence among adolescents and other age groups presents a hugely challenging, multi-faceted, intractable public health problem requiring an approach that fully engages with and responds to its complexity.^7^

Complex systems thinking provides a framework that enables us to account for the numerous spheres of interacting—and often uncertain—influences on obesity.^8^ This is in contrast to more traditional approaches which tend to focus on single or a small number of factors, and treat the linkages between them as linear and predictable. One of the defining features of complex systems is interconnectedness—of people and places, of physical, commercial, political and other environments, of factors such as increasing urbanization and of shifts in working patterns and transport. Another is ‘feedback’, whereby loops of influence across the system show how factors can amplify an undesirable situation or may drive a system to maintain an existing state. For example, fast-food advertising may increase demand, thus increasing supply, which in turn increases demand, which increases profit available for advertising and so on. Intervening in these kinds of feedback loops (FBL) may provide particularly effective ways to achieve change within a system.^9^

A complex systems lens can help account for the interlinked, dynamic, relations between a range of factors.^10^ The system of interest here comprises a set of variables, people, institutions, sectors, contexts and other factors that, in various ways, interact to drive adolescent obesity.^11^^,^^12^ By taking a complex systems approach to obesity, we can conceptualize it as an outcome of many, interdependent factors within a connected whole^13^ and identify ways in which changing these factors might contribute to mitigating the effects they have on dietary and physical activity behaviour of adolescents, and thus on their obesity prevalence.

This article reports findings from an international project called ‘Confronting obesity: Co-creating policy with youth’ (CO-CREATE) which uses a complex systems framework to explore—with young people—the drivers of adolescent obesity and potential policy actions, across five European countries. For the segment of the project reported here, we conducted system mapping sessions using the group model building (GMB) technique, to produce system maps, in the form of causal loop diagrams (CLD). We show commonalities across all countries, represented in the merged system map, which expresses qualitatively, the adolescents’ perceptions of the drivers of obesity and we focus on the key feedback loops (FBLs) in the map, which indicate potential focal points for change.

## Methods

System mapping using GMB is a useful tool for clarifying and helping to generate hypotheses about the connections between the various contributing factors in any given complex problem,^14^ and thereby, to identify potential points in the system to intervene; the map may be used subsequently to develop a computational simulation model.^9^ GMB is a well-recognized way to depict qualitatively the drivers of obesity, and the complexities it entails and to help guide the development of policy responses.^15^^,^^16^

FBLs show how complex behaviours arise from the interactions of the system’s components and the effect these interactions have on the system. They are particularly salient parts of a CLD because they can represent leverage points at which interventions may improve the system’s performance by potentiating or breaking the loops. Additionally, FBLs provide key mechanisms that can be formalized into a simulation model for testing the potential impact that policy options have on the problem at hand.^17^^,^^18^ FBLs are described as either ‘reinforcing’ whereby they amplify the effects of a given set of actions; or ‘balancing’, which act as forces of resistance, eventually limiting growth, maintaining stability or reaching equilibrium. Because the loops may themselves be interlinked and reinforce and/or counteract each other, they can generate an aggregate representation of the problem’s behaviour. The structure of a system is determined by the network of causal FBLs necessary to explain why certain key elements in the system behave over time as they do. FBLs are thereby seen as the ‘engines’ of the model.

Working with adolescents across five European countries (The Netherlands, Norway, Poland, Portugal and the UK), we conducted system mapping sessions using a GMB approach, to produce qualitative, diagrammatic illustrations of the perceived drivers of obesity in the form of CLDs. We then merged the maps into one and identified salient FBLs.

### Recruitment

A common protocol with the same recruitment principles was used across all five countries. This was translated into national protocols which were individually designed to fit local contexts. The aim was to recruit 10–15 young people for each group, with 4 groups in each of the 5 countries. The selection of participants included adolescents aged 16–18 years, covering schools across the socio-economic spectrum and across diverse geographic areas; the protocol was adapted, in each country, according to the different ways in which SES is measured and to divergent municipal governance structures. Schools were selected using purposive cluster sampling by SES and all countries followed similar steps in contacting and recruiting schools, who in turn asked students to volunteer to take part in the GMB sessions. We focused on the group characteristics according to broad socio-economic metrics, not individual characteristics. Indeed, participants were not screened for specific characteristics or experiences, as GMB does not seek to depict individuals’ views, rather more broadly those of the group on the issue of concern so participants were not recruited on the basis of specific characteristics or experiences. Consent to take part was obtained from the adolescent participants, and in countries where it is required by law (Poland and Portugal), from their parents/guardians. GMB was done using a format of two 1.5-h sessions with each group although due to timetable restrictions, some schools had one 3-h session instead.

### Group model building

**Figure 1:**
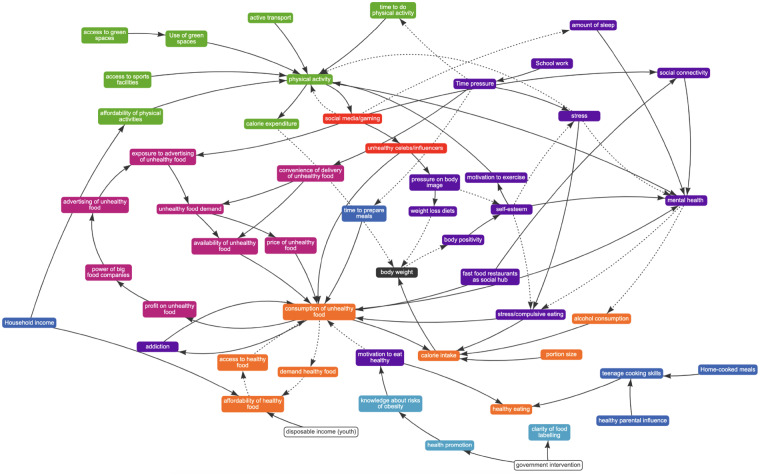
Integrated map representing views of young people in 20 groups in 5 European countries

The method used for generating the system maps was GMB: a structured, collaborative process designed to guide participants through various stages to generate a CLD, which depicts the factors they believe contribute to adolescent obesity.^15^^,^^16^^,^^19^ The sessions follow a scripted routine to guide participants through steps to create the CLD. A CLD shows not just the factors but also the ways in which they may be causally related to each other and to obesity.^20^ The process was carefully structured to take participants through various exercises which result in a CLD (figure 1) that represents a consensual view on the system’s components, relationships and boundaries.^19^

The GMB process that we used^19^^,^^21^ requires multiple roles to facilitate and document a workshop and to generate a digital CLD, for which we used software called STICK-E (Systems Thinking In Community Knowledge Exchange), developed by Deakin University in Melbourne, Australia.^22^ In addition to facilitating the sessions, authors were involved in the various other roles: building the map in STICK-E, taking notes to document the discussion and resulting map. The notes taken form a crucial part of the process after the session, whereby the facilitators go over the CLD, comparing the diagram with the notes, to ensure that the CLD does indeed reflect the discussions that took place.

### Map merging

To create a ‘master’ CLD representing all 20 maps generated by adolescents, a novel process was developed to merge the 4 CLDs from each country into 1, then the 5 country maps into 1 ‘master map’. Because of the variation across the maps, the merging was a pragmatic process conducted by N.S. and A.A. following a protocol developed at Deakin, informed by principles and examples laid out in existing system dynamics literature,^15^^,^^19^^,^^23^ checked by all other facilitator-authors for validity; firstly, each CLD was edited to remove variables that had connections going only in or only out. The second step in merging the four CLDs was to select the one with the most variables remaining as the ‘base map’. Subsequently, each variable on the other CLDs was examined in relation to the base and judged to be either: discarded as a duplicate; added to the base; or not fitting anywhere. Some variables were discarded entirely if they did not fit the sense of another part of any CLD or were placed to the side on the base for later deliberation. The rough ‘base map’ was then scoured to ensure links from the feeder CLDs were correctly represented, to check no variables had been unnecessarily discarded and to ensure that the final CLD reflected clearly the causal relationships suggested by participants. The same process was followed to merge the five country maps into the final ‘master map’.

### FBL identification

**Figure 2:**
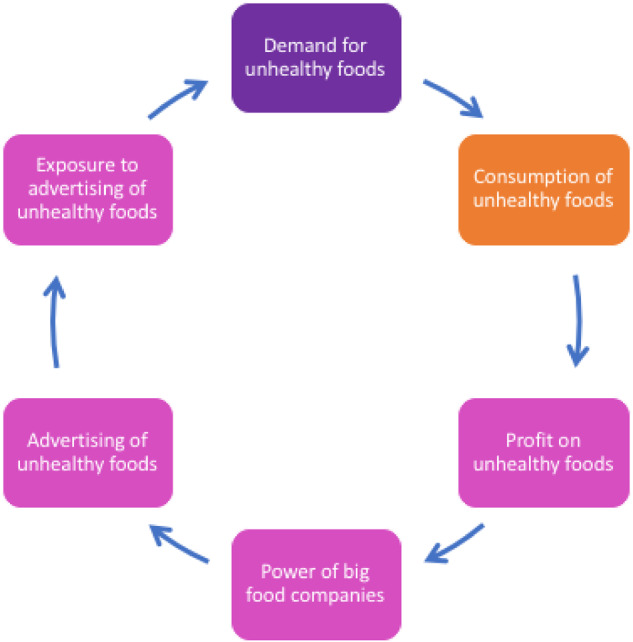
Commercial drivers of adolescents’ unhealthy diet

**Figure 3:**
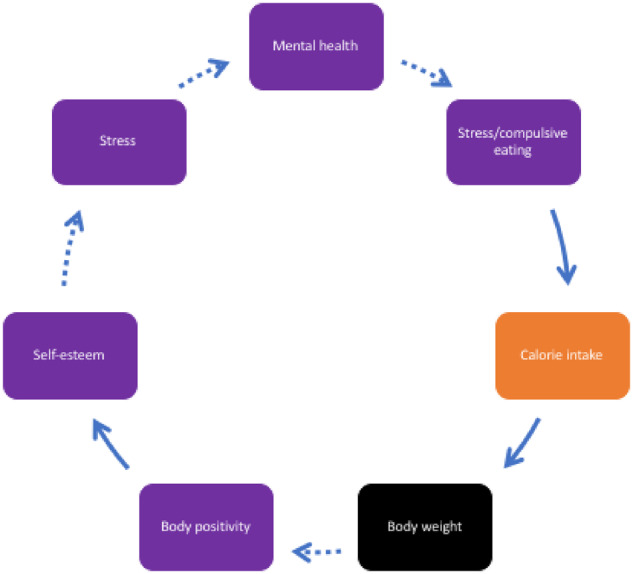
Mental health and unhealthy diet

**Figure 4:**
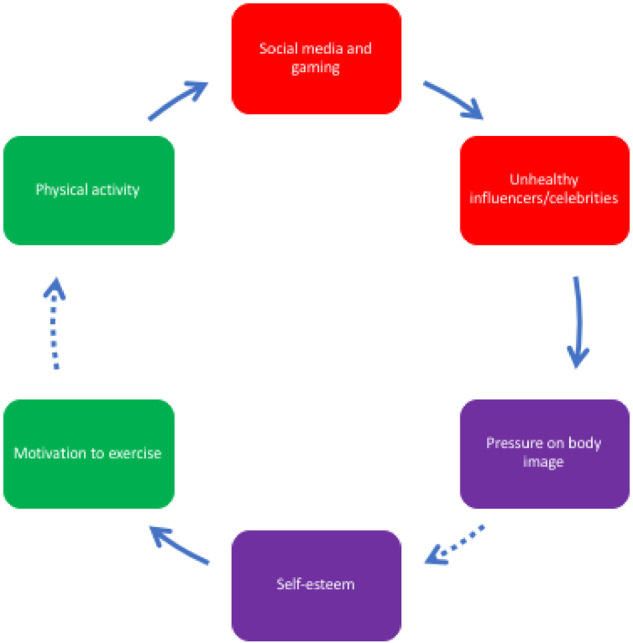
Social media use among adolescents, their body image and motivation to exercise

Once the map merging was complete, FBLs were then highlighted within the ‘master’ CLD. Three FBLs that demonstrate key themes in the CLD, and that offer opportunities for policy intervention, are presented in figures 2–4.

## Results

We secured the participation of schools through the support of colleagues via various networks and municipal authorities. Thus, 257 students, aged 16–18 years, at 18 schools across 5 countries participated in 20 separate school-based system mapping processes. The nature of GMB is such that the focus is on generating the diagram as a group, so we did not take further demographic characteristics of the individual participants such as exact age or gender. Each group created 1 CLD; the 20 maps were amalgamated following the merging protocol.

The variables on the CLD were broadly colour-coded into themes: emotional pressure and time, online activity, physical activity, food and drink intake, economic/commercial influence, knowledge/information and home life with body weight in the centre. The variables are linked using solid lines to illustrate positive relationship and dotted lines to demonstrate negative correlation (table 1). The resulting merged map in figure 1 illustrates a summary of the 257 participating adolescents’ views of the drivers of obesity. Though it is usual GMB practice to validate further the revised map with the people who created it, this was not possible because of time constraints on the part of the schools and young people involved.

**Table 1:** Colour coding for figures

Colour/line	Variable class/relationship
**Purple**	Emotional/mental/pressure/time
**Red**	Online activity/influence
**Green**	Physical activity (or lack of)
**Orange**	Food and drink intake
**Fuschia**	Economic/commercial influence
**Light blue**	Knowledge/information
**Dark blue**	Home life
**Black**	Body weight
**White**	Unclassified
**Solid line**	Positive relationship
**Dotted line**	Negative relationship

As shown in the diagram in figure 1, most groups of young people with whom we created system maps highlighted ubiquitous advertising and access to unhealthy foods and drinks, as well as the low cost of these products, in making them highly accessible. They strongly emphasized the role of stress, anxiety or depression, as well as negative pressures on body image. Low body positivity was usually related to social media influencers (individuals who provide product placements and endorsements in their social media feeds) and celebrities creating unrealistic expectations of what a healthy weight and a ‘beautiful body’ should look like. The adolescent participants spoke about stress-, binge- or comfort-eating, excessive intake of unhealthy foods and physical inactivity. One group mentioned the cost of leisure or fitness centres and another raised the point that public transport is often used instead of active transport, all contributing to low levels of physical activity. In terms of the broader societal issues, several students mentioned the role of the food industry and the power it has in producing and promoting processed food.

FBLs in CLDs encapsulate the causal chains that may well be the most important in influencing the issue, in this case, adolescent obesity. Three FBLs particularly stand out in this CLD: commercial drivers of adolescents’ unhealthy diets; adolescent mental health issues as triggers for eating unhealthy food; and social media use among adolescents, related to their body image and motivation to exercise.

Figure 2 depicts the participants’ views of how the commercial food environment—including the influence of large food manufacturers and advertisers—drives the consumption of unhealthy food among adolescents. They discussed how influential big food companies are, as manifested by ubiquitous marketing and advertising of unhealthy foods, which they felt increased their exposure to, and subsequently demand for, these foods. They mentioned advertising in traditional places including billboards and television but also raised the point that advertising is increasingly via online sources and specifically on social media channels. The young people in the GMB sessions described how the power of large food companies is part of the cycle which feeds into unhealthy eating, driving increased profit for such companies.

Participants raised mental health-related eating patterns, citing compulsive, addictive, binge- or comfort-eating, often as a way of coping with stress or other mental health issues. Figure 3 illustrates the perceived role of poor mental health in driving unhealthy diets. Young people reported that excessive body weight and/or poor perception of body weight, can result in poor self-esteem and stress, all of which contributes to worsening mental health. This, they reported, was a contribution to stress-eating of predominantly unhealthy foods, contributing to weight gain and body image issues—hence another FBL.

Indeed, as illustrated in figure 4, body image pressure was raised in relation to the role of influencers and celebrities on social media, and the air-brushing of pictures online, perpetuating distorted ideals of attractiveness and resulting in poor body-image and low self-esteem. Poor self-esteem was reported to reduce willpower to exercise, and largely due to loneliness and increased sedentary behaviour, led to more screen time i.e. another FBL. Screen time was mainly described by participants to consist of playing video games and following social media, where they were further exposed to so-called ‘influencers’.

Figure 4 illustrates the way that the adolescents linked social media use, impact on self-esteem and motivation to engage in physical activity. Influencers were considered to be enormously important in affecting young people’s behaviour. They were considered another form and source of advertising, increasing consumption of unhealthy foods that are sponsored by influencers, thus increasing calorie intake and body weight, reducing body positivity, self-esteem, motivation to exercise and exercise itself. This perpetuates the loop by leading to an increase of social media and gaming (a sedentary behaviour), and further exposure to social media influencers pushing unhealthy foods.

## Discussion

There is increasing agreement across research, policy and practice that obesity results from the complex interplays between multiple social, economic, environmental and biological factors and individual characteristics.^5^^,^^24^ Multi-faceted public health problems such as obesity are increasingly conceptualized using complex systems thinking. The UK Government Foresight report *Tackling Obesities: Future Choices* report,^24^ which described and illustrated obesity as a complex problem, remains a cornerstone of our understanding of obesity globally.

The CLD generated by adolescents, reported here, represents novel insights, not only because there has been little research with adolescents about obesity but also because of the systems approach used with them; most research using systems mapping to examine obesity has been with adults, even when addressing childhood obesity. The resulting conceptual frameworks illustrate the key factors expressed by young people as drivers of adolescent obesity; this contrasts with more typical descriptions based on linear relations, for example between unhealthy eating and body weight. By working with adolescents in five European countries to create system maps—CLDs—we have shown what they perceive to be a wide range of determinants of obesity. By showing participants’ views in a systems map, we have been able to demonstrate causal chains driving dietary and physical activity habits and identified feedback loops within the maps. These FBLs offer up potential focal areas for effective interventions.

An additional value of this research was to expose the role in obesity of factors such as social media and mental health, which were consistently highlighted by the young people with whom we worked with but are not well reflected in research and policy. For example, a recent Cochrane review of interventions for preventing obesity in children^25^ only included randomized controlled trials of diet and/or physical activity interventions for preventing overweight or obesity in children 0–17 years. No other factors which could drive excess weight in this age group were included explicitly. Social media and influencers do not figure widely in the published literature on obesity. ‘Screen time’ and ‘physical activity’ have been investigated,^6^ and our study extends this work by examining FBLs involving these factors, which have considerable implications for public health policies and actions.

There are, however, limitations to the results in this article as in any qualitative research: they reflect the views of the participants and cannot claim to be fully representative of the general population. The sampling process for the GMB was designed to be representative at the school/group level and discussions with leaders/teachers encouraged offering the opportunity to the widest possible range of individuals within each cohort. Details of individual characteristics were not recorded in this community-based approach and neither were participants screened for other factors such as eating disorders. It is, therefore, possible that the CLD are skewed by biases of the participants, given that they ultimately self-selected for participation. For example, it is known that adolescent girls are more likely to have low self-esteem and body dissatisfaction than boys, while screen time tends to be higher amongst boys and all these factors are linked to dietary behaviours.^26^^,^^27^ Additionally, the merged system map reflects views that were broadly held across groups, so some proposals that came only from a very small number of participants were not included. For example, although heavier bodies were broadly linked to lower self-esteem, a few participants pointed out that body positivity movements had, in some cases, promoted acceptance of and thereby self-worth associated with larger bodies.

Using GMB, we created system maps of drivers of adolescent obesity as perceived by 20 groups of 16–18 years old as part of the CO-CREATE project. GMB sits within the ‘system dynamics’ tradition—it is designed such that the maps can be used not just qualitatively as described here but also to feed into system dynamics simulation models. CO-CREATE will build on this mapping work by taking a complex systems approach to adolescent obesity throughout the project. The maps represented here will be used in subsequent workstreams: one in which adolescents will work in ‘Youth Alliances’ to examine the FBLs to determine where policy action could be taken to engender change, and another, in ‘Dialogue Forums’ in which youth will discuss the feasibility of their policy ideas with policy-makers and other stakeholders. The maps will also be used to inform the development of a system dynamics computer model to simulate the potential outcomes of such policy actions. The CO-CREATE project demonstrates the inclusion of young people in the formation and development of public health initiatives that affect them.

This research used GMB to explore young people’s perceptions of the drivers of adolescent obesity in five European countries. In doing so, it has not only revealed participants’ views on the determinants of diet and physical activity but also, situated them within a system. GMB with young people demonstrates a workable way of involving young people in research and a starting point for finding ways to develop effective policy actions that resonate with them. Moreover, the approach helps serve as a tool to shift the paradigm from individual-level, linear and predictive models to complex system approaches that account for social, economic, political and other drivers of obesity.
